# COVID-19 Infection and Guillain-Barre Syndrome: A Case Series

**DOI:** 10.7759/cureus.21998

**Published:** 2022-02-07

**Authors:** Kendal Carpenter, Ayman Iqbal, Romil Singh, Keerti Deepika, Thoyaja Koritala, Nitesh Jain, Ram Sanjeev Alur, Ramesh Adhikari, Vishwas S Mellekate

**Affiliations:** 1 Neurology, Union Health, Terre Haute, USA; 2 Internal Medicine, Dow Medical College, Dow University of Health Sciences, Karachi, PAK; 3 Critical Care, Allegheny Health Network, Pittsburgh, USA; 4 Health Equity and Inclusion, Nemours Alfred I. duPont Hospital for Children, Wilmington, USA; 5 Internal Medicine, Mayo Clinic, Mankato, USA; 6 Pulmonary and Critical Care Medicine, Mayo Clinic, Mankato, USA; 7 Internal Medicine, Marion Veterans Affairs Medical Center, Marion, USA; 8 Hospital Medicine, Franciscan Health, Lafayette, USA; 9 Geriatrics, Brown University, Providence, USA; 10 Neurology, Indiana University School of Medicine, Terre Haute, USA

**Keywords:** albuminocytologic dissociation, neurological complication, gullian-barre syndrome, cytokine storm, acute inflammatory demyelinating polyradiculoneuropathy, covid-19, myasthenia gravis

## Abstract

The coronavirus disease 2019 (COVID-19) pandemic brought about an unprecedented time. Multiple systemic complications have been recognized with the severe acute respiratory syndrome coronavirus 2 (SARS-CoV-2) as it can do much more than affect the respiratory system. One of the intriguing neurological complications is Guillain-Barre syndrome (GBS). We reviewed three cases in which patients presented with GBS following COVID-19 infection. All three cases had positive lumbar puncture results with albumino-cytological dissociation. Each patient was treated with plasmapheresis and improved clinically. Although an exact causal relationship between COVID-19 and GBS cannot be drawn from this case series alone, it signifies the importance of this complication. It warrants further studies to establish the causal relationship. One should have a high suspicion for acute inflammatory demyelinating polyneuropathy (AIDP) in patients presenting with acute onset of ascending weakness following COVID-19 infection.

## Introduction

Since the pandemic began, much has been discovered about the severe acute respiratory syndrome coronavirus 2 (SARS-CoV-2). Although primarily affecting the respiratory system, coronavirus disease 2019 (COVID-19) has been shown to cause several neurological complications and symptoms, including stroke, dizziness, headache, encephalopathy, hypogeusia, and hyposmia [1]. 

In two-thirds of cases, Guillain-Barre syndrome (GBS) is an autoimmune inflammatory disease affecting the nerve roots and peripheral nerves, preceded by a recent infection [2]. There have been many reported cases of GBS following COVID-19 infection, showing a possible association between the two entities. Other viruses known to trigger GBS include cytomegalovirus, Epstein-Barr, influenza, Zika, and Hepatitis E virus [3]. GBS occurs through an immune-mediated response targeting nerve tissue [4]. It is characterized by acute onset of ascending motor and sensory deficits [5]. Deep tendon reflexes are typically diminished to absent. It is a heterogeneous disorder and has several variants, including acute inflammatory demyelinating polyradiculoneuropathy (AIDP), acute motor-sensory axonal neuropathy (AMSAN), acute motor axonal neuropathy (AMAN), Miller Fisher syndrome (MFS), and acute panautonomic neuropathy [6]. AIDP is widely referred to as the “classic” subtype [6]. 

Herein we present three patients at a community hospital who presented with signs and symptoms consistent with GBS following COVID-19 infection.

## Case presentation

Patient 1

A 55-year-old male with acute onset of bilateral hand and feet numbness and tingling sensations presented to the hospital. The patient stated he had new-onset difficulty with walking and fear of falling. His symptoms had started about two weeks back and progressed. Initially, it was paresthesia and then gradually developed weakness. He states his weakness was in both feet and then in the entire lower extremities. The patient has poorly controlled diabetes mellitus type 2 (hemoglobin A1C - 11.2%). On initial examination, the patient also had overlapping features of peripheral neuropathy, noted to have impaired crude touch, vibratory sense, proprioception, and pinprick sensation to bilateral feet. The patient was taking gabapentin at the time of presentation. His initial presentation was attributed to peripheral neuropathy secondary to diabetes mellitus, and he was discharged with a plan to follow up as an outpatient for further management. The patient had no symptoms suggesting COVID-19 infection. After a week, he was readmitted with a worsening bilateral lower extremity weakness and multiple falls. The patient's family -- wife and son had COVID-19 four weeks before admission. He admitted to intermittent subjective fevers over the past few weeks but was never tested for COVID-19. The patient was admitted with worsening bilateral lower extremity weakness and frequent falls. On examination, the patient had bilateral lower extremity weakness with distal weakness more significant than proximal weakness, absent deep tendon reflexes, and decreased lateral spinothalamic and proprioception sensory loss in bilateral lower extremities. MRI cervical and lumbar spine showed degenerative disc disease and neural foraminal narrowing at multiple levels; there were no spinal cord signal abnormalities and no other acute pathology to explain the patient's symptoms.

Electromyography and nerve conduction studies showed severe motor neuropathy, predominantly axonal, with features of demyelinating neuropathy. These features were concerning for a variant of GBS-AMSAN (acute motor and sensory axonal neuropathy). The cerebrospinal fluid (CSF) analysis showed elevated protein at 103 mg/dL with no cells. The patient's nerve conduction studies (NCS) were suggestive of AMSAN, and CSF findings were consistent with albumino-cytological dissociation; the patient was started on plasmapheresis. Anti-ganglioside antibody testing was negative. The patient received four sessions of plasmapheresis, and strength in bilateral lower extremities improved significantly. He was discharged home and subsequently seen in the Neurology clinic. He showed continued improvement in his motor strength in both lower extremities and could walk without support. 

Patient 2 

A 67-year-old male presented to the hospital with generalized weakness and falls. The patient had been to a family gathering on “Thanksgiving,” and several of those family members tested positive for COVID-19 the next day. Three days later, the patient experienced a high fever with a maximum of 102 degrees Fahrenheit, headache, muscle aches, and generalized weakness. He opted not to get tested for COVID-19. Five to six days later, he started noticing bilateral lower extremity weakness and fell to the extent that he needed to borrow his girlfriend's walker to ambulate. On examination, the patient had a generalized weakness, worse in bilateral lower extremities, and absent deep tendon reflexes throughout. His weakness was more significant in distal lower extremities than in proximal lower extremities. On the second day of admission, the patient complained of difficulty swallowing and perioral numbness. He was subsequently transferred to the intensive care unit (ICU) for close monitoring of his respiratory status. The patient did not consent for electromyography (EMG)/NCS and lumbar puncture for CSF analysis. The patient was started on plasmapheresis due to great clinical concern for GBS. The patient agreed to have a lumbar puncture after a session of plasmapheresis. The CSF results showed elevated protein at 415 mg/dL with no cells. A total of five sessions of plasma exchange was completed, and his weakness improved. After a significant improvement in his strength, the patient was discharged from the hospital. 

Patient 3

A 46-year-old female presented to the hospital with a chief complaint of subjective feeling of left-sided weakness. Her weakness was gradual in onset and progression. It had started about three to four days back and was progressive, which brought her to the emergency room (ER). The patient was diagnosed with COVID-19 two months before the onset of weakness, and she was asymptomatic. She had no other significant comorbidities. On examination, the patient was noted to have weakness in all four extremities, and lower extremities were more affected than upper extremities. The MRI of the brain and cervical spine did not show any acute pathology. The CSF analysis revealed elevated protein at 111 mg/dL with no cells, raising the concern for GBS. Plasmapheresis was done two days after hospitalization due to initial suspicion of stroke due to left side weakness and further work up-MRI of the brain was done to rule out stroke and CSF analysis was done for diagnosis of GBS. The patient underwent five sessions, and the patient's weakness improved.

## Discussion

Much has been learned about the SARS-CoV-2 virus since the pandemic first began. Several neurological complications have been reported, studied, and thought to occur via two different pathways. The first proposed mechanism, hematogenous (infection of leukocytes or endothelial cells) or trans-neuronal route (olfactory or other cranial nerves) of dissemination, causes complications such as hyposmia and hypogeusia in COVID-19 patients. The second proposed mechanism is an abnormal immune-mediated response induced by a cytokine storm that can lead to severe neurological complications such as GBS, encephalomyelitis, and encephalitis [2]. 

The SARS-CoV-2 invades epithelial cells by binding with angiotensin-converting enzyme (ACE-2) receptors, localized inflammation, endothelial activation, tissue damage, and dysregulated cytokine release [7]. Cytokine storm starts with the secretion of vascular endothelial growth factor, monocyte chemoattractant protein-1, IL-1, IL-2, IL-6, IL-8, IL-10, tumor necrosis factor-a, interferon-g, and reduced E-cadherin expression on epithelial cells. Increased levels of cytokines and chemokines are associated with the severity of COVID-19 [7]. The cytokine storm associated with COVID-19 infection can also worsen other neurological conditions like myasthenia gravis -- autoimmune neuromuscular disorder, culminating in myasthenic crisis and poor clinical outcomes [8].

Although primarily a clinical diagnosis, a high index of suspicion for GBS is necessary. Further confirmatory testing of lumbar puncture, electrodiagnostic studies, and antibody testing is essential. It is typical in GBS to have albumino-cytological dissociation in which there is elevated protein with normal cell count [5]. Electrodiagnostic studies will further aid in diagnosis and help in differentiating between subtypes. However, it is essential to note that electrodiagnostic studies may be normal during early symptom onset and will not show disease patterns for two to three weeks [5]. Typical findings on electrodiagnostic studies include prolonged or abnormal F wave, absent H reflex, reduced motor conduction velocities, and absent or diminished sensory action potentials [9]. Electromyography/Nerve conduction studies (EMG/NCS) will show a demyelination pattern in AIDP. As the name implies, electrodiagnostics will reveal axonal degeneration of motor nerves with no sensory involvement. AMSAN consists of axonal degeneration of both sensory and motor nerves. 

Gangliosides are found on the myelin of the peripheral nerves. In GBS, an immune-mediated response can be triggered that results in antibodies against the gangliosides [10]. These autoantibodies are more frequently recognized in specific subtypes of GBS. AMAN is often anti-GM1 and anti-GD1A antibody positive but has also been associated with anti-GAINac-GD1a and anti-GD1b antibodies [11]. Anti-GQ1b antibodies are present in over 95% of cases of the Miller Fisher variant of GBS [6]. Testing for anti-ganglioside antibodies can certainly aid in diagnosing GBS, especially if suspecting MFS. However, a negative test result does not exclude GBS diagnosis [4]. 

Treatment of GBS typically consists of immunomodulatory therapy with intravenous immune globulin (IVIG) or plasma exchange (PE) and supportive treatment. Both IVIG and PE are equally efficacious in aiding in recovery. The decision between these two options is dependent on individual circumstances such as cost and availability [6]. During treatment, the patient should be closely monitored for signs of respiratory distress and include the use of lung parameters. The patient is also watched for evidence of cough or dysphagia. Muscle strength should certainly be tested to monitor evidence of improvement or progression. Around 85% of patients will return to baseline in six to twelve months. Several factors that can indicate a poor prognosis include age, severity of symptoms, rapid disease progression, and prolonged mechanical ventilation [9]. Supportive therapy will consist of continued rehabilitation with physical therapy, treatment of neuropathic pain, and psychological stress [5]. 

A study conducted in Northern Italy showed a significantly higher number of GBS patients during the COVID-19 outbreak and a high frequency of GBS patients with COVID-19, thus highlighting the possible role of SARS-CoV-2 in triggering GBS [12].

There are certain limitations associated with Case 1. Firstly, he did not get tested for COVID-19; therefore, we cannot definitively state that he had the virus. However, given his high fever and close contact with individuals known to be infected, it is highly likely that the patient had COVID-19. Secondly, evaluation for weakness is compounded by his lumbar and cervical radiculopathy, which might have also contributed to his bilateral leg weakness. Moreover, his diminished deep tendon reflexes (DTRs) could also be an exam finding from his uncontrolled diabetic peripheral neuropathy and lumbar radiculopathy. However, lumbar puncture and EMG results support GBS diagnosis and could not be explained by the lumbar/cervical radiculopathy or peripheral neuropathy. 

Case 2 is also limited because the patient did not receive formal testing for COVID-19. Then again, as in Case 1, his clinical symptoms and close contact with the infected family raise high suspicion that he had the infection. This patient had a very classic case of GBS with the rapid onset of ascending weakness, including the development of respiratory distress, albumino-cytological dissociation in CSF studies, and rapid improvement following plasmapheresis. 

Case 3 initial presentation was not as typical for GBS as the other two cases. Her weakness was diffuse, and any acute onset of weakness was masked because she was pretty deconditioned following several months of unrelated illness and multiple hospital admissions before our evaluation. However, the CSF studies and improvement in weakness following plasmapheresis highly support the diagnosis of GBS. Anti-ganglioside antibody testing was negative in Case 1. Due to high clinical suspicion and CSF findings -- albumino-cytological dissociation, in cases 2 and 3, we did not do anti-ganglioside antibody testing. Though there were case reports of patients with COVID-19 and GBS, we found one reported case with positive GM1 ganglioside and COVID-19 in the literature, and the rest of them were either not tested or not positive for GM1. The pathophysiology of COVID-19 related GBS and MFS may be significantly different from cases in the pre-pandemic era though the clinical presentation is similar [13].

Interestingly, a UK-based cohort study revealed no significant relationship between GBS and COVID-19 infection. No differences were seen in clinical and neurophysiological characteristics, the severity of GBS between COVID-19 and non-COVID-19 subjects. According to this study, no scientific data support the molecular mimicry association between SARS-CoV-2 to GBS at nucleic acid level [14]. 

Although the strength of the relationship between COVID-19 and GBS is uncertain, with a mere association that cannot amount to etiology, these case studies presented here indicate that GBS can be a neurological complication following infection with the COVID-19 virus. All three cases were preceded by COVID-19 infection confirmed by electrodiagnostic evidence of demyelinating and axonal neuropathy, CSF with albumino-cytological dissociation, and clinical improvement following plasmapheresis. This case series suggests that differential diagnosis should include GBS if a patient presents with acute onset of weakness following COVID-19 infection. 

The observed alterations in the SARS-CoV-2 Delta variant due to variations in spike protein resulted in a novel Omicron variant; the transmission rate of the omicron variant is exponential [15]. The ongoing pandemic continues to wreak havoc with new variants. It is worth mentioning that during the pandemic, when catering to patients with neurological symptoms, doctors should entertain SARS-CoV-2 infection as a differential diagnosis to avoid misdiagnosis and diagnostic delays [16].

## Conclusions

COVID-19 is a relatively new disease, its entire spectrum is unknown, and the virus, despite immunizations, is rapidly evolving. Therefore, in GBS cases in which no preceding infection or root cause is identified, it would also be prudent to perform COVID-19 polymerase chain reaction (PCR), COVID-19 antibody testing, and detailed clinical history. Additional research involving larger population studies needs to be conducted to determine the exact relationship between COVID-19 and GBS.
